# Soft Sensor-Based Monitoring and Efficient Control Strategies of Biomass Concentration for Continuous Cultures of *Haloferax mediterranei* and Their Application to an Industrial Production Chain

**DOI:** 10.3390/microorganisms7120648

**Published:** 2019-12-04

**Authors:** Thomas Mainka, Nicole Mahler, Christoph Herwig, Stefan Pflügl

**Affiliations:** 1 TU Wien, Institute for Chemical, Environmental and Bioscience Engineering, Gumpendorfer Straße 1a, 1060 Vienna, Austria; thomas.mainka@tuwien.ac.at (T.M.); Nicole.Mahler@morgansindall.ch (N.M.); christoph.herwig@tuwien.ac.at (C.H.); 2 Competence Center CHASE GmbH, Altenbergerstraße 69, 4040 Linz, Austria; 3 Kompetenzzentrum Holz GmbH, Altenbergerstraße 69, 4040 Linz, Austria; 4 CD Laboratory on Mechanistic and Physiological Methods for Improved Bioprocesses, Gumpendorfer Straße 1a, 1060 Vienna, Austria

**Keywords:** continuous bioprocessing with cell retention, soft sensor-based process monitoring, halophiles, bioremediation, *Haloferax mediterranei*

## Abstract

Continuous bioprocessing using cell retention allows the achievement of high space-time yields for slow-growing organisms such as halophiles. However, the lack of efficient methods for monitoring and control limits the application of biotechnological processes in the industry. The aim of this study was to implement a control and online monitoring strategy for biomass in continuous cultures. For the first time, a feedforward cultivation strategy in a membrane-based cell retention system allowed to control the biomass concentration of the extreme halophilic *Haloferax mediterranei* at defined levels. Moreover, soft sensor-based biomass estimation allowed reliable monitoring of biomass online. Application of the combined monitoring and control strategy using industrial process water containing formate, phenol, aniline and 4,4′-methylenedianiline could for the first time demonstrate high throughput degradation in this extremophilic bioremediation process, obtaining degradation efficiencies of up to 100%. This process demonstrates the usefulness of continuous halophilic cultures in a circular economy application.

## 1. Introduction

Many areas of industrial production result in the continuous generation of large quantities of complex process water streams. Often, they require (pre)treatment, either chemically or biologically, before they can be released to the environment, which is necessary if the process water contains toxic, hazardous, or inhibitory contaminants with detrimental effects on the environment and public health [1,2,3,4,5]. There are several industrial sectors producing saline process water streams, for which halotolerant microorganisms offer a sustainable alternative for their treatment [6,7]. 

Halophilic microorganisms have huge potential in terms of waste water treatment since they can be cultivated under non-sterile conditions and are able to use a broad variety of carbon sources [8,9,10]. Furthermore, they are also able to degrade a large variety of contaminants in process waters, e.g., aromatic compounds like phenol or aniline [11,12].

On the other side, drawbacks of halophilic processes are low specific growth rates, rendering them unsuitable for high throughput processes as required for industrial process water treatment applications. In addition, biomass as the catalyst is the most critical process parameter in a biological remediation process and process performance (i.e., degradation efficiency) is directly proportional to the number of biocatalysts in the reactor. Therefore, a process for treatment of large quantities of industrial process water would have to meet three major criteria: (1) Simple control of biomass concentration, (2) accurate online monitoring of biomass concentration, and (3) high productivity to achieve high liquid throughput. 

To establish a robust bioprocess that meets the defined requirements, continuous bioprocessing is necessary. However, due to low specific growth rates, a conventional chemostat process with halophiles can only be operated at low dilution rates. A chemostat fermentation system can be extended with a cell retention system, retaining a controlled number of cells inside the reactor [13,14]. This allows decoupling the liquid dilution rate from the specific growth rate of the culture (i.e., the biomass dilution rate) according to the retention rate and as a result higher dilution rates can be reached. The retention rate, or recycle ratio, describes the ratio of cell-free filtrate-flow from the reactor to the input feed-flow [14,15,16]. In conclusion, a cell retention set-up allows to adjust biomass concentrations independent from the specific growth rates and therefore allows to establish a process with high biomass concentrations and high liquid dilution rates. 

Nevertheless, only suitable online monitoring of biomass concentration allows its accurate control. There are numerous concepts that work on the online determination of the process parameters like biomass concentration—among them, optical, capacitance, and calorimetric methods [17]. An overview of the advantages and disadvantages is given elsewhere [18]. Moreover, mathematical models or soft sensors could be an appropriate solution for describing and monitoring complex bioprocess variables (i.e., biomass) and help to control process states [19,20,21]. Soft sensors are a combination of a “software” and a “sensor”, meaning signals are provided online by a sensor and are evaluated with mathematical models [20,22]. For calculating the biomass concentration in a bioreactor, soft sensors using balances for carbon and the degree of reduction have already been investigated previously [23,24]. However, for the approach in this study, existing models were extended for continuous systems with cell retention.

The aim of this study was the first implementation of a feedforward control strategy combined with an online monitoring concept for the biomass concentration for cultivations with the extreme halophilic *Haloferax mediterranei*. To achieve this, a lab-scale bioreactor was extended with a membrane to perform cell retention bioprocessing. For the control of biomass concentration, the parameters retention rate *R* and substrate concentration in feed *s_in_* were used. The feedforward control loop was closed with a soft sensor-based tool for the online state estimation of biomass concentration. Therefore, existing principles of online biomass estimations using off-gas measurements and elemental balances were adapted to a continuous cell retention process and the performance compared to offline measurements.

Using either synthetic process water or real industrial process water with the organic contaminants formate, aniline, phenol, and 4,4′-methylenedianiline (MDA), the feasibility of both the control and monitoring concept could be demonstrated. Based on these results, the extreme halophilic archaeon *Haloferax mediterranei* was used for the first time in a high throughput treatment process to remove four organic contaminants from an industrial process water. This shows successful use of halophilic continuous cultures in a circular economy application, where an industrial process water after biological purification can be reused for further purposes (e.g., NaCl-containing process water for chlorine production), thus combining chemical and biological processing. 

## 2. Material and Methods

### 2.1. Bioreactor Setup

**Figure 1:**
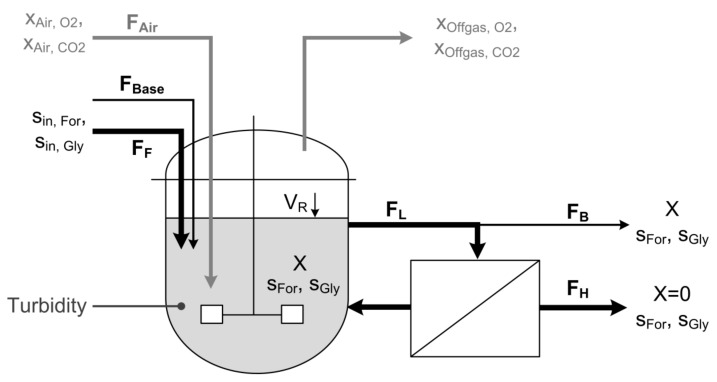
Scheme of the cell retention setup. A constant feed (F_F_) supplies the cells with fresh substrate and media components. Base (F_Base_) is added to hold the pH on a constant level of 7.0. A pump continuously circulates the cell suspension as loop flow (F_L_) through the membrane module to separate cell-free harvest (F_H_). Bleed flow (F_B_) is continuously removed to eliminate cells and sustain steady state conditions. To guarantee a constant reactor volume (V_R_) flows for Feed, Base, Harvest and Bleed have to meet the following equation: F_F_ + F_Base_ = F_H_ + F_B_. Biomass is monitored using a turbidity probe and a soft sensor that is driven by measurements of off-gas composition.

A schematic diagram of the experimental setup is shown in Figure 1. Continuous cultures with and without cell retention were performed in a corrosion-resistant stirred tank reactor with a working volume of 2.3 L (PEEK Labfors bioreactor, Infors, Switzerland), equipped with a 420 cm^2^ microfiltration unit (model: CFP-2-E-4A, polysulfone membrane, 0.2 µm pore size, GE Healthcare, Germany). Peristaltic pumps were used for loop (Ecoline, Ismatec, Germany), feed, bleed, and harvest (Lambda Preciflow, Lambda Instruments, Switzerland). The dilution rate was kept constant at 0.1 h^−1^ for all experiments. The pH was measured using an Easyferm probe (Hamilton, Switzerland) and adjusted using 0.5 M NaOH via the integrated dosing system of the Labfors system. Reactor volume was kept constant at 1 ± 0.05 L. The reactor volume, feed, base, and acid consumption are continuously monitored by laboratory scales with 0.1 g resolution (Mettler Toledo, Columbus, OH, USA). The inlet airflow was kept constant at 100 mL min^−1^ via a mass flow controller (Brooks Instrument, Hatfield, PA, USA). Dissolved oxygen was measured using an Oxyferm probe (Hamilton, Switzerland) and kept above 20% to guarantee aerobic conditions in the reactor. Oxygen transfer was adjusted by variation of stirrer speed between 400 and 560 rpm. Composition of off-gas was determined using a BlueSens gas analyzer system (BCP O_2_ and CO_2_, BlueSens, Germany). To reduce its water content the off-gas passed a countercurrent condenser before entering the gas analyzer system. 

A corrosion-resistant turbidity probe (InPro8050, Mettler Toledo, Columbus, OH, USA) was used for online measurement of biomass concentration. The probe was calibrated to Nephelometric Turbidity Units (NTU) using Formazin calibration standards (Sigma Aldrich, St. Louis, MO, USA). The probe is equipped with an infrared-LED to beam light at 880 nm via a fiber optic cable into the liquid medium. Backscattered light is captured and led back to the transmitter via a fiber optic cable where it is processed as turbidity signal. 

The online data monitoring and process control were executed with a process information management system (Lucullus, SecureCell, Switzerland).

### 2.2. Strain and Medium

*Haloferax mediterranei* DSM 1411 was obtained from DSMZ (Deutsche Sammlung für Mikroorganismen und Zellkultur, Braunschweig, Germany). Prior to continuous culture, batch cultivation was carried out in the bioreactor to reach an initial biomass concentration of 3 g L^−1^. All cultivations were performed at a temperature of 37 °C and at pH 7.0. Synthetic medium contained (g L^−1^): NaCl 150; NH_4_Cl 1.5; KH_2_PO_4_ 0.15; FeCl_3_ 0.005; MgCl_2_ · 6 H_2_O 1.3; MgSO_4_ · 7 H_2_O 1.1; CaCl_2_ · 2 H_2_O 0.55; KCl 1.66; Trace elements solution 1 mL [(mg/100 mL): FeSO_4_ · 7 H_2_O 139; CuSO_4_ · 5 H_2_O 100; MnCl_2_ · 7 H_2_O 120; CoCl_2_ · 2 H_2_O 44; ZnSO_4_ · 7 H_2_O 86]. Glycerol was added as a substrate in a concentration of 1–4 g L^−1^. 

For the industrial medium, process water from polyurethane production of an industrial partner was used. It contained 150 g L^−1^ NaCl and formate as an organic contaminant in a concentration range of 200 ± 20 mg L^−1^. For the industrial medium, the process water was supplemented with mineral media components (except NaCl) equivalent to the synthetic medium and glycerol to final concentrations of 2–10 g L^−1^ as indicated. The elemental composition of *H. mediterranei* grown in continuous cultivation on glycerol was determined by Universität Wien, Institute of Physical Chemistry [25]. The elemental composition of the *H. mediterranei* biomass on the substrate glycerol was CH_1.57_O_0.63_N_0.13_P_0.02_S_0.01_ with a molar mass M_X_ of 26.4 g mol^−1^ [26]. The cell concentration was estimated by the measurement of the optical density at 600 nm (OD_600_). In case absorption exceeded OD_600_ 0.5 the samples were diluted with a saline solution (150 g L^−1^ NaCl) to prevent lysis of the cells. Correlation of OD_600_ with biomass concentration was calculated as described in the literature [26]: Biomass (g L^−1^) = 0.48 · OD_600_.

### 2.3. Continuous Culture and Feedforward Control Concept

The steady state condition in biological systems with cell retention is determined by the dilution rate *D* and the retention rate *R*. The parameters are calculated as follows from the feed flow *F_F_*, the base flow *F_Base_*, the harvest flow *F_H_*, the bleed Flow *F_B_* and the reactor volume *V_R_*:

(1) $$D=\frac{F_{F}+F_{Base}}{V_{R}}$$

(2) $$\;R=\frac{F_{H}}{F_{F}+F_{Base}}=\frac{\left(F_{F}+F_{Base}\right)-F_{B}}{F_{F}+F_{Base}}\;$$

(3) $$\;F_{F}+F_{Base}=F_{B}+F_{H}\;$$

Calculation of the volumetric flow rates *F_F_, F_B_, F_H_*, and *F_Base_* was based on the change of balance signals over time. In a steady state system, the biomass concentration for cell retention reactors depends on the biomass yield *Y_X/S_*, the substrate consumption (s_in_-s) and the retention rate *R* (see Equation (4)). Therefore, the set point for the biomass concentration *X_Set_* can be calculated as stated in Equation (5). The dilution factor *f_D_*, thereby, includes the dilution caused by base addition (see Equation (5)). In the case of carbon-limited conditions, Equation (5) further can be simplified as *s* is set to zero. In a setup with cell retention, the specific growth rate *µ* is depending on the retention rate R and the dilution rate *D* (see Equation (6)).

(4) $$X_{Steady}=\frac{Y_{X/S}\cdot (s_{in}-s)*f_{D}}{\left(1-R\right)}$$

(5) $$\;f_{D}=\frac{F_{F}}{F_{F}+F_{Base}}\;$$

(6) $$\;{µ}_{Set}=\left(1-R\right)\cdot D\;$$

### 2.4. Soft Sensor Rate Calculation and Statistical Test for Consistency

Soft sensors for physiological rate calculations are already described in the literature [23,24,27]. The biomass formation rate *r_X_* is calculated by means of a redundant equation system (degree of redundancy of 1) comprising the Carbon balance (C balance) and Degree of Reduction balance (DoR balance) (see Equations (7) and (8)):

- Carbon Balance:
  (7) $$Fr_{S,\;For}+r_{S,\;Gly}=CER+r_{X}$$
- Degree of Reduction Balance:
  (8) $$\gamma_{For}\cdot r_{S,\;For}+\gamma_{Gly}\cdot r_{S,\;Gly}+\gamma_{O2}\cdot OUR=\gamma_{X}\cdot r_{X}$$

where r_S_ is the volumetric substrate uptake rate for formate and glycerol, calculated according to Equation (9); *CER* is the carbon dioxide evolution rate, calculated from off-gas composition according to Equation (10); *OUR* is the oxygen uptake rate, calculated from the off-gas composition according to Equation (11); and *γ* is the degree of reduction of the component indicated in the index. *F_Air_* is the airflow into the reactor as determined by the mass flow controller, the different x are the molar fractions of CO_2_, O_2_, or H_2_O, and V_mol_ is the molar standard volume. The term *r_inert_* compensates the proportion of gaseous water x_H20_ in the off-gas (Equation (12)). All rates are referred to the number of carbon molecules with the objective of obtaining rates with a unit of C-mol h^−1^. The aromatic compounds aniline, phenol, and MDA were not taken into account for the balances since they only contribute a maximum of 2% of the total carbon in the system.

(9) $$r_{S}=F_{F}\cdot (s_{in}-s)$$

(10) $$\;CER=\frac{F_{Air}\cdot r_{inert}\cdot \left(x_{CO2_{Offgas}}-x_{CO2_{Air,\;}}\right)}{V_{mol}}\;$$

(11) $$\;OUR=\frac{F_{Air}\cdot r_{inert}\cdot \left(x_{O2_{Air}}-x_{O2_{Offgas}}\right)}{V_{mol}}\;$$

(12) $$\;r_{inert}=\frac{1-x_{O2_{Air}}-x_{CO2_{Air}}}{1-x_{O2}-x_{CO2}-x_{H2O}}\;$$

The biomass turnover rate *r_X_* is calculated by data reconciliation of elemental balances. For this purpose, Equations (8) and (9) are transformed in matrix form, which described elsewhere in detail [27]. 

To check the consistency of the model, a test function (χ^2^ distribution) is used. Degree of freedom of χ^2^ distribution equals the degree of redundancy of the equation system (here: 1) and the confidence level is set to 95%. For these parameters, the threshold for the statistical test value *h* is 3.84, i.e., with a probability of 95% the test value *h* is to be expected in the range of 0 to 3.84. For *h* values >3.84, consistency of the equation system is statistically rejected. For these time points, an outlier in soft sensor calculations might be indicated. A step-by-step explanation for calculation of *r_X_*, as well as a more in-depth description of this consistency check can be found elsewhere [24,27,28,29]. However, the used formulas can be found the Supplementary Materials. All calculations were performed with the software MATLAB (2018b, MathWorks, Natick, MA, USA).

### 2.5. Soft Sensor Biomass Estimation 

Biomass concentration *X_SteadyState_* during steady state conditions is calculated according to Equation (4), by using the estimated biomass turnover rate *r_X_* obtained from data reconciliation.

**Figure 2:**
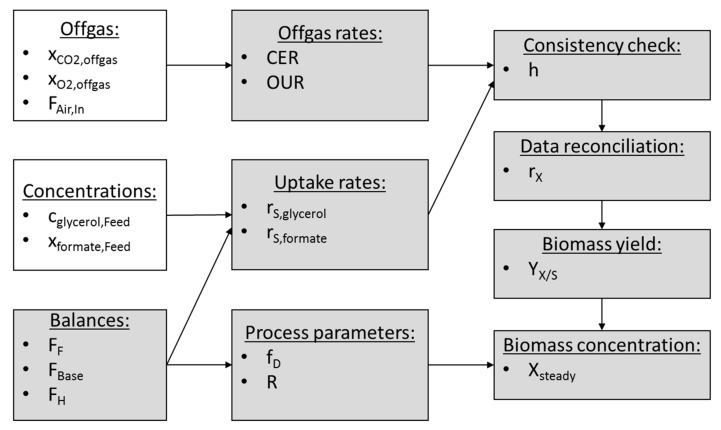
Soft sensor workflow. The calculation of the biomass concentration at steady state conditions in a bioreactor with a cell retention system is carried out according to this workflow. White boxes represent measured values and grey boxes represent calculated variables.

Online estimation of the biomass conversion rate *r_X_* enables online estimation of parameters that are crucial for the assessment of the metabolic state. In Figure 2, a schematic overview of the soft sensor workflow to calculate the biomass concentration is shown. For the data reconciliation in order to calculate *r_X_*, the balances described in Equations (8) and (9) are used. 

### 2.6. Analytical Procedures

Substrate quantification in the feed and harvest samples was done as described previously [25], using HPLC (Vanquish UHPLC systems, Thermo-Fisher, Waltham, MA, USA) with an Aminex HPX-87H column (Bio-Rad, USA) at 60 °C, an isocratic eluent of 4 mM sulfuric acid in Milli-Q water with a flow of 0.6 mL min^−1^, followed by UV detection at 210 nm and RI detection (RefracoMax520, ERC, Germany). In brief, samples were centrifuged and the supernatant was diluted (1:10) with 40 mM sulfuric acid before 10 µL was injected in the HPLC. The samples were analyzed for residual formate and glycerol, as well as the formation of organic acids. The standards, used for quantifications, were prepared the same way as the samples and diluted with 40 mM sulfuric acid.

For the quantification of aromatic compounds in feed and harvest samples, a reversed-phase HPLC measurement (Vanquish UHPLC systems, Thermo-Fisher, Waltham, MA, USA) was carried out, using an Acclaim^TM^ PolarAdvantage column (Thermo Scientific, Waltham, MA, USA, C16, 3 µm, 120 Å, 4.6 × 150 mm) at 30 °C. Aromatic compounds were detected using a UV detector at 210 nm. The flow was 1 mL min^−1^ with a gradient system (0–2.5 min: 5% A, 95% B; 2.5–5 min: 25% A, 75% B; 5–20 min: Linear decrease of B from 75% to 30%, rest A). After each measurement, a washing step was carried out (0–5.5 min: Linear increase of C from 75% to 95%, rest A; 5.5–15 min: 5% A, 95% C; 15–20 min: 5% A, 95% B). Eluents were: (A) acetonitrile; (B) 25 mM KH_2_PO_4_ (pH 3.5 with 1 M H_3_PO_4_); and C) MiliQ water. Samples were centrifuged prior to analysis and 10 µL undiluted supernatant was injected for HPLC analysis. 

## 3. Results and Discussion

### 3.1. Development of a Biomass Control and Monitoring Concept on Synthetic Medium

In order to develop a control strategy that enables the control of biomass concentration of extremely halophilic *H. mediterranei*, a continuous membrane-based cell retention system was used. A feedforward biomass control strategy was successfully developed on synthetic medium using glycerol as a cheap and widely available carbon source [30,31,32]. Furthermore, a biomass online monitoring tool was established to calculate biomass concentrations in steady state experiments.

Moreover, formate as additional carbon source was also tested as the control concept strategy should later be transferred to real industrial process water contaminated with formate. Previous experiments showed that *H. mediterranei* could not grow when cultivated on formate as sole carbon source (data not shown), but is degraded in the presence of glycerol. 

The feedforward control concept was established by varying the parameters *R* and s_gly_ to obtain different biomass concentrations (Table 1). Doubling the substrate concentration in the feed leads to a two-fold increase of the steady state biomass concentration in the reactor. This can clearly be seen when comparing experiments 1 and 2, where the glycerol concentration in the feed was decreased from 4 g L^−1^ to 2 g L^−1^ resulting in a decrease of biomass concentration from 8.4 g L^−1^ to 4.1 g L^−1^. This correlation is known from chemostat cultivations without cell retention. Additionally, by varying the retention rate R, the Bleed:Feed ratio (1-R) is also changed. An increase in R results in a lower ratio, meaning that less biomass is removed from the reactor via the bleed stream. As a direct result, decreasing the Bleed:Feed ratio by 50 % while also decreasing s_gly_ by 50 % the same overall biomass concentration reactor should be obtained. This can be seen when comparing experiments 2 and 3. Both aim for the same biomass concentration of 4.1 g L^−1^, but the Bleed:Feed ratio for experiment 3 was only half compared to experiment 2. By additionally decreasing the glycerol concentration by 50%, the same biomass concentration was achieved (Table 1). For estimation of the variables r_X_, r_S_, X_soft sensor_, and Y_X/S_ in Table 1, data reconciliation and steady state equations as described in Material & Methods was used.

To check for potential effects on biomass estimations by the soft sensor formate was added in a concentration of 300 mg L^−1^ in experiments 2* and 3* of the experimental design (Table 1). Formate was completely degraded in all experiments (data not shown) and the functionality of the soft sensor-based method was not influenced by additional formate in the medium.

**Figure 3:**
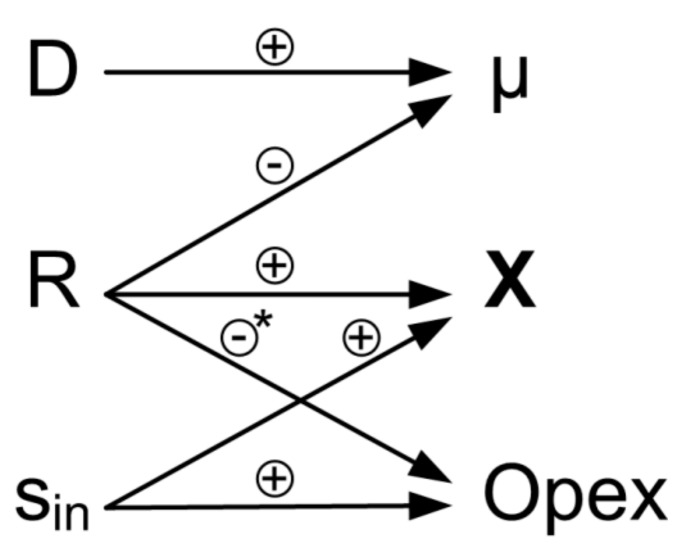
Elements and interactions of the process control concept. Control variables (dilution rate D, retention rate R and substrate concentration s_in_) are used to vary process variables (specific growth rate *µ*, biomass concentration *X*) and operational expenditures (Opex). Positive correlation indicated with (+), negative correlation indicated with (−). * Influence of R on Opex is highly dependent on the process. A negative correlation can be seen when biomass has to be disposed of (e.g., for industrial process water treatment or extracellular products). When a product is intracellular, a high bleed stream is desirable.

An overview of the elements of the process control concept and their high interactivity is shown in Figure 3. Using a cell retention system allows to obtain high biomass concentrations while maintaining low substrate concentrations in the feed. This has a beneficial effect on the operational expenditures of the process (Opex), whereas high values of *R* result in low bleed streams and lower costs for disposal of redundant biomass. Hence, high retention rates are usually preferred in cell retention systems. The control concept uses the self-regulation mechanisms in continuous culture. Disturbing factors, e.g., temporary change in feed composition, lead to disturbance of biomass growth. However, in case the biomass concentration has not been decreased below a critical threshold, the culture will reach a steady state again, according to the settings of *D* and *R*.

### 3.2. Application of the Feedforward Control Concept on Industrial Medium

**Figure 4:**
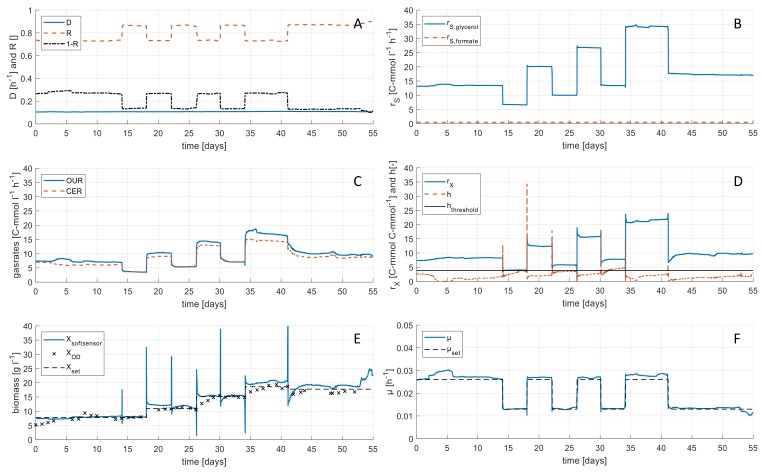
Rate calculations during continuous cultures of *H. mediterranei* with cell retention using real industrial brine. (**A**) Dilution rate *D*, retention rate *R*, and Bleed:Feed ratio (1-R) calculated from online balance data. (**B**) Substrate consumption rate *r_S_* calculated from feed rate and substrate concentrations. (**C**) Oxygen uptake rate (OUR) and carbon dioxide evolution rate (CER) calculated from online off-gas data. (**D**) Biomass turnover rate *r_X_* calculated by the soft sensor from reconciled data and the statistical test value h for soft sensor calculation. Additionally the threshold for h of 3.84 is marked as black line. (**E**) Biomass concentration calculated from the soft sensor and from OD values. Root-mean-square deviation (RMSD) of soft sensor measurements in comparison to biomass set points was determined to be 2.0 g L^−1^. (**F**) Specific growth rate *µ* calculated with biomass turnover rate and biomass concentration from soft sensor.

The process knowledge gained in the experiments on the synthetic medium was applied for continuous remediation of industrial brine containing 200 mg L^−1^ formate by *H. mediterranei*. In a 1300 h continuous process, biomass concentration was controlled stepwise to reach four different levels in the range of 5 to 20 g L^−1^, while the dilution rate D was held constant for the entire duration of the experiment. The phases were held constant for a minimum of 96 h to guarantee that the culture reaches steady state conditions (equivalent to 9.6 volume changes). Analysis of harvest samples showed C-limited conditions at all time points. Each step was performed at two different specific growth rate levels (0.013 and 0.026 h^−1^) to investigate the influence of µ on the yields (O2, CO2, and biomass). The variation of R, Bleed:Feed ratio (1-R), and rS over time can be seen in Figure 4A,B. Carbon dioxide evolution rate (CER) and oxygen uptake rate (OUR) as inputs for the monitoring tool are shown in Figure 4C. Using the soft sensor, the biomass formation rate rX was calculated (see Figure 4D). In Figure 4D, also the statistical test value h can be seen. It indicates the consistency of the soft sensor calculations. In almost all time points it was below the threshold of 3.84, where gross errors larger than 10% are not occurring. However, between day 32 and 35 of the continuous experiment, h exceeded the threshold value, indicating that the rates cannot be calculated within a 10% error interval. This might be caused by measurement errors in the gas rates or the substrate concentrations and leads to incorrect calculations. Nevertheless, calculated values for biomass concentration in steady states XS Steady using Equation (4) show good conformity when compared with offline OD measurements as well as with the applied set point for the biomass concentration (see Figure 4E).

Furthermore, the results show that biomass concentration could be held constant on the four different biomass concentration levels despite changing the specific growth rate *µ* on two levels (see Figure 4F). 

**Figure 5:**
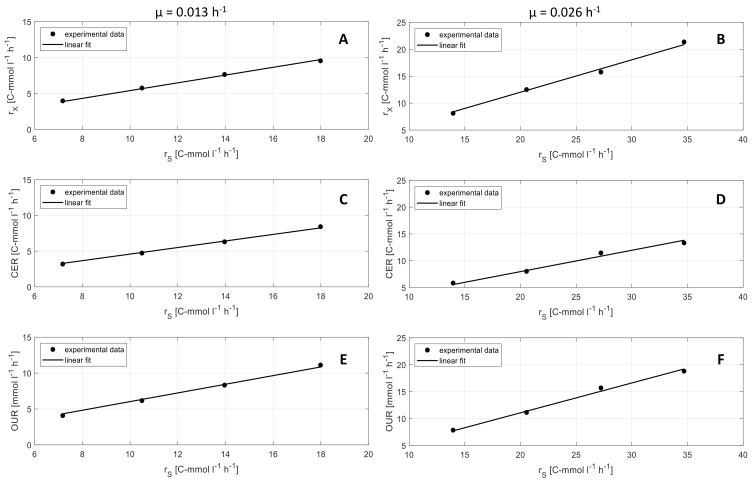
Metabolic yields determined by linear regression. (**A**–**F**) Biomass turnover rate (r_X_), carbon dioxide emission rate (CER) and oxygen uptake rate (OUR) over substrate uptake rate (r_S_) at a two different specific growth rates of (left: 0.013 h^−1^, right: 0.026 h^−1^) (• experimental data, - linear fit).

Physiological rates, that are determined for the soft sensor, offer important additional information in terms of metabolic activities of the cells. Figure 5 shows the correlation of CER, OUR, and *r_X_* with substrate turnover rate *r_S_* in the industrial medium. The results show a linear correlation of all three rates. The slope of the graphs are the yields *Y_CO2/S_, Y_O2/S_*, and *Y_X/S_* (see Table 2). Yields show significant differences according to the specific growth rate *µ*. A higher *µ* (0.026 h^−1^) leads to higher biomass yields Y_X/S_, lower CO_2_ yields Y_CO2/S_, and lower O_2_ yields Y_O2/S_ compared to lower *µ* (0.013 h^−1^). 

A comparison of yields for O_2_, CO_2_, and biomass for synthetic and industrial medium did not show significant differences. Nevertheless, the results show the same effect of *µ* on the yields on both media, synthetic, and industrial. Therefore, it is important to use online determined yields for soft sensor-based biomass calculation instead of predetermined yields, since physiological states can change during a process, according to process parameters and medium.

Since the proposed soft sensor is a direct measurement of metabolic activity, it is independent of changes for process parameters like aeration rate, stirrer speed, or change of flow rates for liquid substrates, and only requires steady state conditions of continuous bioprocesses together with consistent offgas measurements and information on the substrate concentration in the feed. Additionally, undefined amounts of carbon introduced when complex media components are used have to be considered in the calculations for the biomass estimations. In conclusion, the developed monitoring strategy allows estimation of biomass concentration even in the presence of other particles, in colored or turbid media or under circumstances where cells form aggregates [33]. Furthermore, it does not require corrosion sensitive hard sensors. This is in stark contrast to standard procedures for measuring biomass concentration, requiring either corrosion sensitive hard sensors or the need for human interference. The tool uses a redundant measurement system (based on gas analysis), which can be used to check the measurements for gross error [28]. Once established, it is easy to handle and can be applied for a wide range of process parameters (different *µ* and *R*).

### 3.3. Degradation of Aromatic Compounds in Industrial Brine

**Figure 6:**
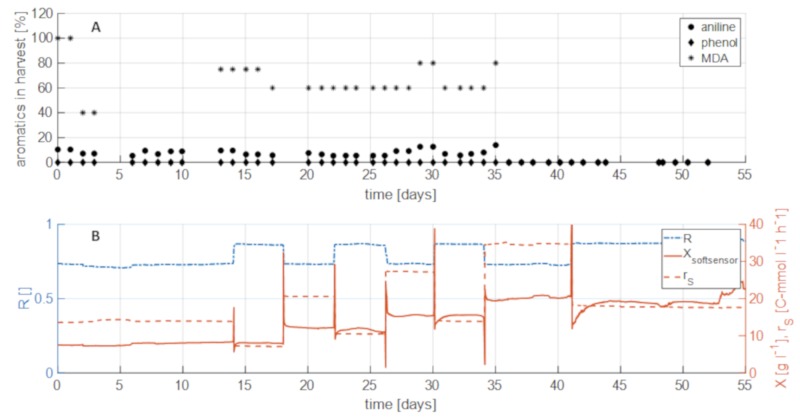
Aromatics in harvest compared with process parameters. (**A**) Share of aromatics left in harvest after treated in bioreactor. Values are calculated according to: Share = aromatics in harvest/aromatics in feed. (**B**) Process parameters X, r_S_, and µ over the process time. Increase of biomass concentration led to complete degradation of aromatics.

After establishing a method for biomass control and monitoring in the membrane-based cell retention system, the ability of such a system to degrade organic pollutants from real industrial process water was evaluated. To that end, different biomass concentrations, specific growth rates and substrate uptake rate were used to study the impact on degradation efficiency of formate and the aromatic compounds phenol, aniline, and MDA contained in different concentrations in the industrial brine. Concentrations of the respective components in the harvest were compared to feed concentrations to determine the degradation efficiency (%, Figure 6A). The individual components were contained in the feed at fixed concentrations and degradation was studied for a period of 1300 h using a continuous process with a dilution rate of 0.1 h^−1^ and different settings for R (and therefore µ) and s_gly_. 

Full formate and phenol degradation was observed throughout the entire process irrespective of the process parameters and biomass concentrations. This was a very interesting finding as it suggests that the degradation efficiency under the conditions tested does not seem to be dependent on a certain biomass concentration or concentration of the individual components. Therefore, high activity of the degradation pathways facilitating degradation of formate and phenol is assumed. 

Degradation of aniline in the industrial process water was slightly less efficient compared to phenol, reaching 80–90% degradation for the time period of 0–35 days. It seems the capacity of the metabolism of *H. mediterranei* for the degradation of aniline is lower than for phenol. Comparing the degradation of aniline with biomass concentration showed that a simple increase of biomass concentration using the feedforward strategy (after day 35) led to complete degradation of aniline. The degradation of aniline in the real brine could therefore be successfully shown if a suitable bioprocessing strategy (in this case high biomass) is utilized. A similar behavior could be observed for the degradation of MDA. At low and intermediate biomass concentrations (<15 g L^−1^) degradation was incomplete at around 20–40% efficiency. Like for aniline, by influencing the biomass concentration as process parameter, also the degradation efficiency of MDA could be increased from 40% to 100%. This indicates a biomass concentration-depending degradation of aniline and MDA, whereas the degradation of phenol seems to be independent from the biomass concentration. Furthermore, it could also be shown that the degradation does not depend on the specific growth rate, since no MDA and aniline were detected at two different settings for *R* and *s_in_* (compare Figure 6B). 

In literature, the pathway of phenol and aniline degradation in halophilic bacteria is described via meta- or ortho-cleavage of catechol [6,34,35]. On the other hand, MDA was reported to be degraded by activated sludge, also in saline waste water [36,37]. However, no degradation pathway for MDA has been reported so far, but it appears likely that the degradation pathway is similar to aniline degradation as MDA can be converted into aniline via cleavage of the methyl bridge between the two aromatic rings [37].

The results show, that the developed feedforward control strategy for biomass concentration works successfully and can be used for degradation of organic contaminants from real industrial brine at high efficiency and productivity. 

Although membrane-based cell retention in bioreactors for continuous cultivation of extremophilic cells has already been reported [38,39,40,41], the current work nicely expands these approaches by using a simple but nevertheless efficient bioprocessing control and monitoring strategy to demonstrate the applicability of extreme halophilic organisms for a circular economy process that requires high throughput degradation of organic compounds, in order to reuse industrial process in further production steps. 

Bioprocesses that are easy to handle and have a robust control strategy are more likely to be integrated into existing production facilities (e.g., in the chemical industry). Therefore, it is envisioned that the current work can contribute to overcome typical reservations against including biotechnological processes in non-biotechnological industries like high effort for sterile handling, time-consuming training of staff and low space-time yields. The feedforward control concept developed in this study provides the process transparency needed for an operation that is safe, robust and easily implementable in an industrial environment.

## 4. Conclusions

To conclude, a feedforward control strategy for a membrane-based cell retention system was combined with a soft sensor-based monitoring tool to control and real-time monitor biomass concentrations of *H. mediterranei*, depending on the retention rate R and the substrate concentration over a broad range of concentrations. This new approach was successfully applied in a high throughput industrial process water treatment process. Moreover, removal of the four organic pollutants formate, phenol, aniline, and MDA at high productivity and degradation efficiency was demonstrated.

The results presented here provide an excellent framework for future applications of halophiles in continuous bioprocesses. 

## Figures and Tables

**Table 1:** Continuous states on the synthetic medium were controlled by different levels of R and s_in_. Addition of formate is indicated with *. For the calculated values r_X_, r_S_, X_soft sensor_, and Y_X/S_, an error of 10% was assumed.

Experiment	R	S_gly_ [g L^−1^]	S_for_ [g L^−1^]	µ_Set_ [h^−1^]	r_X_ [C-mmol L^−1^h^−1^]	r_S_ [C-mmol L^−1^h^−1^]	X_soft sensor_ [g L^−1^]	Y_X/S_ [Cmol Cmol^−1^]
**1**	0.74	4	0	0.026	8.01	12.35	8.4	0.65
**2**	0.74	2	0	0.026	3.83	5.99	4.1	0.64
2 *	0.74	2	0.3	0.026	4.18	6.87	4.2	0.61
**3**	0.87	1	0	0.013	1.84	3.06	4.1	0.60
3 *	0.87	1	0.3	0.013	2.14	3.79	4.7	0.56
**4**	0.74	2.6	0	0.026	4.89	7.98	5.2	0.61
**5**	0.74	1.4	0	0.026	2.66	4.19	3.0	0.63

**Table 2:** Yield coefficients reconciled along C and DoR balance with industrial medium.

	Y_O2/S_ [mol Cmol^−1^]	Y_CO2/S_ [Cmol Cmol^−1^]	Y_X/S_ [Cmol Cmol^−1^]
**µ = 0.013 h^−1^**	0.60 ± 0.01	0.46 ± 0.01	0.54 ± 0.01
**µ = 0.026 h^−1^**	0.55 ± 0.01	0.40 ± 0.01	0.60 ± 0.01

## Supplementary Materials

The following are available online at https://www.mdpi.com/2076-2607/7/12/648/s1.
