# Supplementary material for: Soft Sensor-Based Monitoring and Efficient Control Strategies of Biomass Concentration for Continuous Cultures of Haloferax mediterranei and Their Application to an Industrial Production Chain
Source: Microorganisms. 2019 Dec 4;7(12):648. doi: 10.3390/microorganisms7120648 (PMC6956367; doi:10.3390/microorganisms7120648)
Supplement: Supplementary file 1 [file microorganisms-07-00648-s001.pdf]

Supplementary material

In this file, equations are provided which were used, but not presented in the recent study. Equations A1 to A3 represent the equation system for rate calculation in a matrix form. Due to noise in the measurements a residuals vector ε needs to be introduced (Equation A4). Measurements for DoR and C balance without significant errors should therefore show residuals not statistically different from zero, indicated by the statistical test value *h*, a measure for the deviation of the residuals (Equation A5 and A6). The variance-covariance matrix of the residuals (Φ) is calculated with Equation A6, including the variance-covariance matrix of the rates (Ψ). The errors for the rates were assumed to be 10% (Equation A7).
The biomass yield $Y_{X/S}$ is determined according to Equation A8.
The specific growth rate μ can be calculated online using Equation A9 and yields for the formation of CO_2 and consumption of O_2 is calculated using Equation A10 and A11.

$EW = 0$	(A1)
$E=\begin{pmatrix} 1 & 1 & 0 & -1 & -1 \\ \gamma_{Gly} & \gamma_{For} & \gamma_{O2} & \gamma_{CO2} & -\gamma_X \end{pmatrix}$	(A2)
$W=\begin{pmatrix} r_{S,Gly} \\ r_{S,For} \\ OUR \\ CER \\ r_X \end{pmatrix}$	(A3)
$EW = \varepsilon$	(A4)
$h=\varepsilon^T \Phi^{-1} \varepsilon$	(A5)
$\Phi = E^T \Psi E$	(A6)
$\Psi = \begin{pmatrix} e_{gly} & 0 & 0 & 0 \\ 0 & e_{for} & 0 & 0 \\ 0 & 0 & e_{O2} & 0 \\ 0 & 0 & 0 & e_{CO2} \end{pmatrix} = \begin{pmatrix} 0.1 & 0 & 0 & 0 \\ 0 & 0.1 & 0 & 0 \\ 0 & 0 & 0.1 & 0 \\ 0 & 0 & 0 & 0.1 \end{pmatrix}$	(A7)
$Y_{X/S} = \frac{r_X}{r_S}$	(A8)
$\mu = \frac{r_X}{X \cdot M_X}$	(A9)
$Y_{CO2/S} = \frac{CER}{r_S}$	(A10)

$Y_{O2/S} = \frac{OUR}{r_S}$	(A11)
